# Knowledge, Attitude, and Practice Toward Type 2 Diabetes Mellitus in the Lebanese Population

**DOI:** 10.7759/cureus.82024

**Published:** 2025-04-10

**Authors:** Najib Y Awad, Battoul Fakhry, Imad Baddour, Omar Ismail, Youssef Jamaleddine, Lea Nohra, Ahmad Twainy, Karim Hassan, Joelle Azzi, Mirna N Chahine

**Affiliations:** 1 Faculty of Medical Sciences, Lebanese University, Beirut, LBN; 2 Department of Basic Sciences, Faculty of Medical Sciences, Lebanese University, Beirut, LBN; 3 Department of Research, Foundation-Medical Research Institute, Beirut, LBN

**Keywords:** attitude, knowledge, lebanon, practice, type 2 diabetes mellitus

## Abstract

Background

Type 2 diabetes mellitus (T2DM) is a major cause of morbidity and mortality worldwide. This study aimed to determine the levels of knowledge, attitude, and practice regarding T2DM among Lebanese patients with T2DM compared to the general population.

Methodology

This was a cross-sectional, online-based questionnaire study conducted in Lebanon among patients with T2DM and the general population between July and September 2021. Data collection encompassed sociodemographic characteristics, habits, and personal/family history of T2DM and assessed the levels of knowledge (44 items), attitude (29 items), and practice (16 items) concerning T2DM.

Results

A total of 1,127 participants were included, and 445 participants had clinically diagnosed T2DM. Only 9% of the participants (103 out of 1,127 participants) showed adequate knowledge level regarding T2DM. Higher scores were noted among young (p = 0.048) and employed (p = 0.025) participants, who also had a higher educational level (p = 0.005) and were aware of their own HbA1c level (p = 0.005). Poor attitude was reported in approximately half of T2DM patients (231 out of 445 participants). A better attitude was noticed in participants with a higher T2DM-related knowledge level (p = 0.016) or a diabetic family member (p = 0.03). Concerning practice, 13.3% of responses were deemed adequate (59 out of 445 participants). Higher levels of T2DM-related knowledge (p = 0.001) and education (p = 0.032) were positively correlated with better practice, in contrast to smoking (p < 0.001) and obesity (p = 0.005).

Conclusions

We found a significant knowledge gap and poor attitude and practice regarding diabetes among Lebanese patients with T2DM, emphasizing the need for targeted awareness campaigns.

## Introduction

Type 2 diabetes mellitus (T2DM) is a chronic metabolic disorder characterized by hyperglycemia. Its underlying mechanism involves both insulin resistance and relative insulin deficiency [1]. Over recent years, there has been a sharp increase in T2DM prevalence, particularly in developing countries, owing to a shift in dietary and behavioral patterns toward an increasingly Westernized and urbanized lifestyle [2]. For instance, Saudi Arabia, a developing country, reported 7,661 cases of T2DM per 100,000 people and a disability-adjusted life year (DALY) rate of 623 per 100,000, surpassing the figures recorded in France of 6,843 T2DM cases and a DALY rate of 564 per 100,000 people [2]. Furthermore, T2DM has deleterious effects on the cardiovascular (e.g., myocardial infarction and atherosclerosis), nervous (e.g., peripheral neuropathy), immune (e.g., impaired phagocytosis and delayed wound healing), digestive (e.g., gastroparesis and non-alcoholic steatohepatitis), musculoskeletal (e.g., acanthosis nigricans and diabetic foot ulcer), renal (e.g., diabetic nephropathy and chronic kidney disease), and ocular (e.g., diabetic retinopathy) systems [3,4]. Therefore, T2DM significantly contributes to poor quality of life, increases healthcare utilization, and is a leading global cause of death [5].

In Lebanon, the prevalence of T2DM is significant (17%) [6]. Interestingly, a recent study highlighted that Lebanese patients with diabetes perceived their quality of life as below satisfactory [7]. Furthermore, Costanian et al. found that Lebanese patients with T2DM exhibited suboptimal adherence to treatment and self-care measures, contributing to elevated complication rates [8]. Numerous knowledge, attitude, and practice (KAP) studies conducted in Lebanon and other countries across the Middle East North Africa (MENA) region revealed insufficient knowledge and poor practices among patients with T2DM, and highlighted areas for improvement [9,10]. Moreover, geographical location, along with its associated sociocultural and economic factors, significantly influenced the overall KAP scores among different populations. Studies reported poor results in Jazan, Saudi Arabia [11], while satisfactory outcomes were observed in Bangladesh [12].

In addition to medical treatment, comprehensive management of T2DM entails patient education on dietary considerations and physical activity to optimize metabolic function. Insufficient understanding of disease risk factors and triggers may lead to suboptimal adherence to treatment plans and an underestimation of the seriousness of the condition. In resource-limited settings such as Lebanon, alongside medical interventions, prioritizing awareness campaigns and educational initiatives proves pivotal in curbing the burden of diabetic complications. Therefore, the instauration of KAP studies presents a wide array of benefits related to the development of appropriate educational programs [13] and tailored campaigns that aim to promote an adequate regulation of blood sugar levels, thus mitigating the possible complications that may arise concerning diabetes [4]. For instance, a study in India found that additional information provided to diabetic patients regarding pharmacological and behavioral modifications led to an increase in their corresponding KAP scores and, concomitantly, to a considerable reduction in their HbA1c levels [14].

Although several KAP studies have been conducted in Lebanon, most surveys solely focused on awareness related to diabetes management (e.g., glycemic control and dietary supplements). In addition, all published data highlights gaps in KAP, highlighting the need for a deeper understanding of the extent of these gaps and identifying factors contributing to them. Furthermore, none of the existing studies objectively and thoroughly categorized the level of awareness. Therefore, we aimed to compare the baseline knowledge level about T2DM between diabetic and non-diabetic individuals in the Lebanese population and to assess the attitude and practices specifically within the T2DM group. In addition, we identified sociodemographic and health-related factors influencing these levels. Our survey mainly focused on general disease characteristics, diabetes complications, the psychological effects of living with diabetes, management strategies, essential aspects of diabetes care, preventive measures, healthcare utilization, and lifestyle behaviors such as physical activity and smoking.

## Materials and methods

Ethical considerations

This observational study was granted an Institutional Review Board clearance from the Ethical Committee of Al-Hayat Hospital (Reference number: ETC-12-2021), in accordance with Good Clinical Practice ICH Section 3, and the principles laid down by the 18th World Medical Assembly (Helsinki, 1964) and all applicable amendments. At the beginning of the questionnaire, an informed consent form written in English or Arabic was incorporated and covered the topic and objectives of the study, the expected duration needed to fill out the survey, and the voluntary and confidential aspects of participation.

Study design and population

This cross-sectional study was conducted using an electronic survey (Google Forms) between July and September 2021 to assess KAP toward T2DM among T2DM patients and the general Lebanese population. Eligible patients were ≥18 years old, Lebanese, currently residing in Lebanon, with or without T2DM, and able to understand English or Arabic.

Participants were divided into two groups. The non-T2DM group, which targeted a minimum of 400 randomly chosen participants from the Lebanese population, based on Slovin Formula: n = N/(1 + Ne^2^), where N represents the population, which consisted of 5,261,372 individuals at the time according to the Index Mundi registry, and e represents a p-value of 0.05. However, we received responses from 682 non-T2DM participants, and their responses were included in our study. The T2DM group targeted a minimum of 400 patients with T2DM based on a priori statistical power analysis using GPower 3.1.9.2 software (Heinrich-Heine-Universität, Düsseldorf, Germany) that revealed that a sample size of 400 was enough to attain a statistical power of at least 90% with an alpha error of 5%, balanced on each side, and effect size set to 5%. However, we received and included responses from 445 T2DM patients.

The population was targeted in all eight governorates (Mohafazat) in Lebanon, i.e., Akkar, North, Beirut, Mount Lebanon, Bekaa, Baalbeck-Hermel, Nabatiyeh, and South. However, as the population is unequally distributed, we decided to regroup them into the following five governorates: Beirut, Mount Lebanon, Bekaa (Bekaa and Baalbeck-Hermel), North of Lebanon (North and Akkar), and South of Lebanon (South and Nabatieh).

Data collection tools and procedures

A re-assembled questionnaire, with minor modifications, from previously published studies and scales [2,15,16] using validated questionnaires about the KAP regarding diabetes mellitus was used. The questionnaire required no more than 10 minutes and was available in both English and Arabic (Appendices) in a Google Forms survey. 

The sociodemographic and patient characteristics section included 11 questions in multiple choice or open-ended style covering gender, age group, occupation, marital status, residency, education level, personal monthly income, smoking status, alcohol consumption, presence of T2DM, other medical conditions, and risk factors. Additional questions were asked for patients with T2DM, such as the duration of their diabetes, HbA1C levels, and whether they receive insulin injections.

The knowledge about T2DM section included 44 questions in “Yes,” “No,” and “I don’t know” format assessing patients’ knowledge of T2DM, including its causes, symptoms, risk factors, consequences, and recommended fasting glycemia levels.

Moreover, the T2DM group’s questionnaire comprised two additional parts concerning the participant’s attitude and practice toward diabetes (Appendices). The attitude and practice toward T2DM sections included 29 (Likert-scale) and 16 (Yes, No, I don’t know) questions, respectively, assessing patients’ attitudes and practices related to T2DM management. Topics covered included regular blood glucose monitoring, adherence to a diet plan, blood pressure management, treatment compliance, diabetes control, maintaining a healthy body weight, regular exercise, and routine medical check-ups.

The questionnaire was translated from English to Arabic using the inverted method of Fortin [17]. The authors first translated it from English to Arabic. Then, the Arabic version was translated into English by a healthcare professional/translator to compare the agreement of the instrument. We conducted a content validation of the questionnaire with experts in diabetes mellitus, who reviewed the items and ensured their relevance and appropriateness. In addition, a pre-test was performed among 10 persons who were not part of the sample to validate the understanding and clarity of the questionnaire items. At the end of the pre-test, the questionnaire was modified as necessary.

The majority of our participants (68.23% of the whole population, 50.64% of the non-T2DM group, and 96.18% of the T2DM group) were interviewed via face-to-face interviews or phone calls with our well-trained team, limiting the bias of self-reporting questionnaires.

Patient involvement

Patients were involved in the design and conduct of this research. During the feasibility stage, the priority of the research question, choice of outcome measures, and methods of recruitment were informed by discussions with patients through phone calls or face-to-face interviews.

Data management

To better categorize KAP scores, we adopted the frequently used following Bloom’s cutoff points: 80-100% (good KAP), 60-79% (moderate KAP), and less than 60% (poor KAP) [18]. In this study, we used the median of the scores and a modified Bloom’s cutoff values with the subcategories of “Poor” and “Fair” scores grouped under the category “limited KAP” about cardiovascular disease and subcategories of “Good” and “Excellent” scores grouped under the category of “adequate KAP” about diabetes. These cutoff values were also based on previously published KAP studies [19,20] (Table 1).

**Table 1:** Grading of the knowledge (K), attitude (A), and practice (P) scores about T2DM into the categories of “Limited” and “Adequate” and the sub-categories of “Poor,” “Fair,” “Good,” and “Excellent.”

Categories	Sub-categories	Knowledge		Attitude		Practice	
		Score (44)	%	Score (145)	%	Score (16)	%
Limited	Poor	≤28	≤63.63	≤101	≤69.65	≤11	≤68.75
	Fair	29–34	65.90–77.27	102–115	70.34–79.31	12–13	75.00–81.25
Adequate	Good	35–39	79.54–88.63	116–130	80.00–89.65	14–15	87.50–93.75
	Excellent	40–44	90.90–100	131–145	90.34–100	16	100

Data analysis

Data analysis was performed using SPSS version 25 (IBM Corp., Armonk, NY, USA). Scores of KAP were computed. As such, 44 items were included for the knowledge score, 29 for the attitude score, and 16 items for the practice score. Sections of the knowledge and practice scores were scored by assigning to each answer “1” if correct and “0” if deemed wrong. Regarding the attitude section, a five-point Likert scale was adopted where “strongly disagree” (if wrong answer) was given 1 point and “strongly agree” (if correct answer) was given 5 points. The overall KAP scores were calculated from the sum of the points granted, where the cut-off value was the median for each section. A reliability test was performed to validate each of the KAP scores.

Descriptive analysis was conducted to represent the variables. Categorical variables were presented by their frequency and percentage. Continuous variables were represented by mean, standard deviation, minimum, and maximum. The Kolmogorov-Smirnov normality test was used to assess the normality distribution of the score. Bivariate analysis, using the Mann-Whitney test, was conducted to test the difference between the non-T2DM group and the T2DM group in terms of the K score. In addition, Kruskal-Wallis test and Spearman correlation test were conducted to assess the factors affecting each of the three KAP scores in the T2DM group. Finally, a multivariate analysis was conducted to test factors affecting each of the three scores in the T2DM group. The significance level was set at 5%.

## Results

Patients’ general characteristics

Of the 1,152 patients who participated in our study, we excluded 25 patients diagnosed with type 1 diabetes mellitus. Of the 1,127 participants included in the final analysis, 445 (39.48%) were diabetic, and 682 (60.52%) did not have diabetes and served as controls. The majority of participants were female (57.1%; 644 out of 1,127 participants). All participants’ overall mean age (SD) was 45.28 (±19.52) years. Compared to the control group, patients with T2DM were older, had higher BMI, and were more likely to be obese (p < 0.001). There was no significant difference in participation rates in each governorate between the two groups. Compared to the control group, participants with T2DM had lower educational levels (p < 0.0001) and higher unemployment rates (p = 0.001) (Table 2).

**Table 2:** General characteristics of patients with T2DM versus controls. T2DM: type 2 diabetes mellitus; SD: standard deviation

		Study groups		Total	P-value
		Control	T2DM		
Age	Mean (SD)	35.15 (16.23)	60.81 (12.75)	45.28 (19.52)	<0.001
	Median	30	60	47.0	
	Minimum–Maximum	18–90	20–90	18–90	
Age	<40 years	443	21	464	<0.001
		65.0%	4.7%	41.2%	
	40–60 years	171	184	355	
		25.1%	41.3%	31.5%	
	60–80 years	59	206	265	
		8.7%	46.3%	23.5%	
	>80 years	9	34	43	
		1.3%	7.6%	3.8%	
Gender	Female (%)	60.4%	51.9%	57.1%	0.003
Weight	Mean (SD)	71.37 (16.54)	80.32 (15.58)	74.90 (16.74)	<0.001
	Median	70.0	80.0	74.0	
	Minimum–Maximum	39–175	45–173	39–175	
Body mass index	Mean (SD)	25.75 (10.58)	29.03 (9.55)	27.05 (10.30)	<0.001
	Median	24.55	29.03 [9.55]	26.00	
	Minimum–Maximum	14.69–177.51	15.57–196.20	14.69–196.20	
Obesity	Non-obese	568	284	852	<0.001
		83.4%	63.8%	75.6%	
	Obese	114	161	275	
		16.6%	36.2%	24.4%	
Governorate	Beirut	58	47	105	0.705
		8.5%	10.6%	9.3%	
	Mount Lebanon	227	145	372	
		33.3%	32.6%	33.0%	
	North Lebanon	187	110	297	
		27.4%	24.7%	26.4%	
	South Lebanon	95	65	160	
		13.9%	14.6%	14.2%	
	Beqaa	115	78	193	
		16.9%	17.5%	17.1%	
Educational level	I did not go to school	3	33	36	<0.001
		0.4%	7.4%	3.2%	
	Primary school	51	195	246	
		7.5%	43.8%	21.8%	
	Bachelor degree	380	184	564	
		55.7%	41.3%	50.0%	
	Master degree	139	23	162	
		20.4%	5.2%	14.4%	
	Doctorate/PhD	32	9	41	
		4.7%	2.0%	3.6%	
	Medical degree	77	1	78	
		11.3%	0.2%	6.9%	
Working status	I do not work	322	257	579	0.001
		47.2%	57.8%	51.4%	
	I currently work	360	188	548	
		52.8%	42.2%	48.6%	
Healthcare professional	No	593	430	1,023	<0.001
		87.0%	96.6%	90.8%	
	Yes	89	15	104	
		13.0%	3.4%	9.2%	

Patients’ clinical characteristics

Out of the 445 patients with T2DM, 40.9% were smokers (182 out of 445), which was significantly higher than the smoking rate in the control group (23.5%; 160 out of 682 participants) (p < 0.001). Regarding alcohol consumption, participants with T2DM were less likely to drink alcohol occasionally in contrast to controls (18.4 vs. 24.6%, respectively, p = 0.046). Hypertension was the most common comorbidity in patients with T2DM, and its prevalence was higher in this group compared to the control group (63.6 vs. 11.7%, respectively, p < 0.001) (Table 3).

**Table 3:** Comorbidities and lifestyle behavior of participants with T2DM versus controls T2DM: type 2 diabetes mellitus

		Study groups		Total	P-value
		Control	T2DM		
Do you have other family members who are diabetic? (first degree: father, mother, full siblings, child)	No	432	115	547	<0.001
		63.3%	25.8%	48.5%	
	Yes	250	330	580	
		36.7%	74.2%	51.5%	
Do you have family members who are diabetic? (second degree: uncles, aunts, nephews, nieces, grandparents, grandchildren, half siblings, and double cousins)	No	263	179	442	0.577
		38.6%	40.2%	39.2%	
	Yes	419	266	685	
		61.4%	59.8%	60.8%	
Do you smoke?	No	493	214	707	<0.001
		72.3%	48.1%	62.7%	
	Yes	160	182	342	
		23.5%	40.9%	30.3%	
	Ex-smoker	29	49	78	
		4.3%	11.0%	6.9%	
Do you drink alcohol?	No	499	354	853	0.046
		73.2%	79.6%	75.7%	
	Yes, occasionally	168	82	250	
		24.6%	18.4%	22.2%	
	Yes, regularly	15	9	24	
		2.2%	2.0%	2.1%	
Hypertension	No	602	162	764	<0.001
		88.3%	36.4%	67.8%	
	Yes	80	283	363	
		11.7%	63.6%	32.2%	
Cardiovascular diseases other than hypertension (e.g., heart failure, coronary artery disease)	No	656	278	934	<0.001
		96.2%	62.5%	82.9%	
	Yes	26	167	193	
		3.8%	37.5%	17.1%	
Cancer	No	677	431	1,108	0.002
		99.3%	96.9%	98.3%	
	Yes	5	14	19	
		0.7%	3.1%	1.7%	
Chronic lung disease (e.g., chronic obstructive pulmonary disease, asthma)	No	659	398	1,057	<0.001
		96.6%	89.4%	93.8%	
	Yes	23	47	70	
		3.4%	10.6%	6.2%	
Renal failure	No	674	415	1,089	<0.001
		98.8%	93.3%	96.6%	
	Yes	8	30	38	
		1.2%	6.7%	3.4%	
Other diseases	No	591	320	911	<0.001
		86.7%	71.9%	80.8%	
	Yes	91	125	216	
		13.3%	28.1%	19.2%	

Around 44% of patients with T2DM (195 out of 445) were diagnosed with T2DM less than 10 years ago. Insulin injections were used by 22.9% of our participants (102 out of 445) with T2DM. In addition, around 80% knew their HbA1c level (355 out of 445 participants), with 40% of the participants (142 out of 355 participants) having HbA1c levels of below 6.4% (Table 4).

**Table 4:** Glycemic control and treatment patterns in patients with T2DM. T2DM: type 2 diabetes mellitus

		Frequency	Percent
Do you suffer from diabetes mellitus?	No	682	60.52
	Yes	445	39.48
	Total	1,127	100.0
Do you take insulin for diabetes?	No	343	77.1
	Yes	102	22.9
	Total	445	100.0
Do you know what your HbA1c level is?	No	90	20.2
	Yes	355	79.8
	Total	445	100.0
What is your HbA1c level?	Less than 5.7%	32	9.0
	5.7–6.4%	110	31.0
	6.4–7%	82	23.1
	7–8%	71	20.0
	More than 8%	60	16.9
	Total	355	100.0
For how many years have you been diabetic?	<5 years	94	21.1
	5–10 years	101	22.7
	10–15 years	86	19.3
	15–20 years	48	10.8
	20–25 years	52	11.7
	25–30 years	19	4.3
	30–35 years	27	6.1
	35–40 years	5	1.1
	40–45 years	8	1.8
	45–50 years	2	0.4
	50–55 years	3	0.7
	Total	445	100.0

Knowledge assessment of the control and diabetic participants

**Figure 1:**
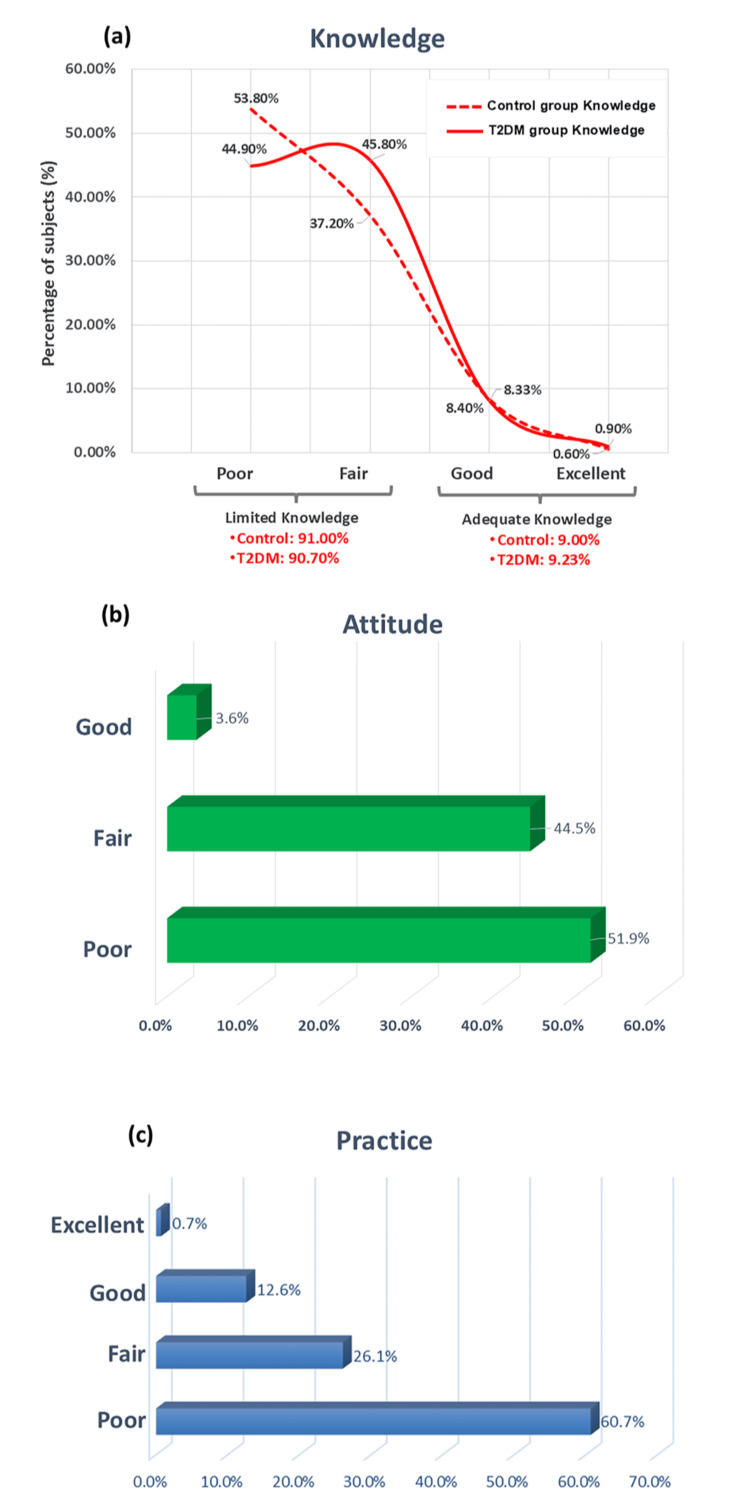
Percentage of participants based on their levels of knowledge (a), attitude (b), and practice (c) regarding T2DM. The knowledge level is assessed in both the T2DM group (N = 445) and the control group (N = 682), while attitude and practice levels are evaluated only in the T2DM group (N = 445). Knowledge levels are categorized as Limited or Adequate, with subcategories of Poor, Fair, Good, and Excellent. In contrast, attitude and practice levels are represented only by subcategories. T2DM: type 2 diabetes mellitus

The knowledge score about T2DM was significantly higher in the T2DM group (28.51 ± 5.15 over 44 (64.80%), N = 445) versus the control group (27.40 ± 5.97 over 44 (62.27%), N = 682, p < 0.001). Around 50% of our participants (567 of 1,127 participants) had poor knowledge about T2DM, and only 9% (102 of 1127) had adequate (Good and Excellent) knowledge (Figure 1, Panel a).

For instance, 71.78% of participants (809 of 1,127) knew that the usual cause of diabetes is a lack of effective insulin in the body. However, only 31.68% (357 out of 1,127) knew that diabetes was not caused by the failure of the kidneys to keep sugar out of the urine. In addition, only 34.16% (385 out of 1,127 participants) did not know where insulin is produced (Table 5).

**Table 5:** Distribution of participants according to their general knowledge concerning T2DM (N = 1,127). T2DM: type 2 diabetes mellitus

Knowledge items		Correct answer	Groups				Total (N = 1,127)	
			Control (N = 682)		T2DM (N = 445)			
			N	%	N	%	N	%
The usual cause of diabetes is the lack of effective insulin in the body	No		51	7.50%	24	5.40%	75	6.65%
	Yes	x	527	77.30%	282	63.40%	809	71.78%
	I don’t know		104	15.20%	139	31.20%	243	21.56%
Diabetes is caused by the failure of the kidneys to keep sugar out of the urine	No	x	233	34.20%	124	27.90%	357	31.68%
	Yes		177	26.00%	92	20.70%	269	23.87%
	I don’t know		272	39.90%	229	51.50%	501	44.45%
Kidneys produce insulin	No	x	392	57.50%	197	44.30%	589	52.26%
	Yes		94	13.80%	59	13.30%	153	13.58%
	I don’t know		196	28.70%	189	42.50%	385	34.16%
In untreated diabetes, the amount of sugar in the blood usually increases	No		17	2.50%	17	3.80%	34	3.02%
	Yes	x	594	87.10%	393	88.30%	987	87.58%
	I don’t know		71	10.40%	35	7.90%	106	9.41%
If I am diabetic, my children have a higher chance of being diabetic	No		57	8.40%	56	12.60%	113	10.03%
	Yes	x	565	82.80%	336	75.50%	901	79.95%
	I don’t know		60	8.80%	53	11.90%	113	10.03%
Diabetes can be cured	No	x	303	44.40%	269	60.40%	572	50.75%
	Yes		314	46.00%	118	26.50%	432	38.33%
	I don’t know		65	9.50%	58	13.00%	123	10.91%
A fasting blood sugar level of 210 is too high	No		27	4.00%	46	10.30%	73	6.48%
	Yes	x	467	68.50%	335	75.30%	802	71.16%
	I don’t know		188	27.60%	64	14.40%	252	22.36%
Regular exercises will increase the need for insulin or other diabetic medications	No	x	362	53.10%	242	54.40%	604	53.59%
	Yes		76	11.10%	54	12.10%	130	11.54%
	I don’t know		244	35.80%	149	33.50%	393	34.87%
There are two main types of diabetes: type 1 (insulin­dependent) and Type2 (non-insulin dependent)	No		27	4.00%	15	3.40%	42	3.73%
	Yes	x	483	70.80%	321	72.10%	804	71.34%
	I don’t know		172	25.20%	109	24.50%	281	24.93%
Medication is more important than diet and exercise to control my diabetes	No	x	399	58.50%	229	51.50%	628	55.72%
	Yes		189	27.70%	189	42.50%	378	33.54%
	I don’t know		94	13.80%	27	6.10%	121	10.74%
Diabetes often causes poor circulation	No		62	9.10%	56	12.60%	118	10.47%
	Yes	x	355	52.10%	218	49.00%	573	50.84%
	I don’t know		265	38.90%	171	38.40%	436	38.69%
Wounds heal more slowly in diabetic patients	No		46	6.70%	45	10.10%	91	8.07%
	Yes	x	562	82.40%	364	81.80%	926	82.17%
	I don’t know		74	10.90%	36	8.10%	110	9.76%
Diabetics should take extra care when cutting their toe nails	No		25	3.70%	21	4.70%	46	4.08%
	Yes	x	497	72.90%	375	84.30%	872	77.37%
	I don’t know		160	23.50%	49	11.00%	209	18.54%
A person with diabetes should cleanse a cut with iodine and alcohol	No	x	47	6.90%	36	8.10%	83	7.36%
	Yes		405	59.40%	332	74.60%	737	65.39%
	I don’t know		230	33.70%	77	17.30%	307	27.24%
The way I prepare my food is as important as the foods I eat	No		104	15.20%	105	23.60%	209	18.54%
	Yes	x	451	66.10%	276	62.00%	727	64.51%
	I don’t know		127	18.60%	64	14.40%	191	16.95%
Diabetes can damage my kidneys	No		24	3.50%	17	3.80%	41	3.64%
	Yes	x	520	76.20%	353	79.30%	873	77.46%
	I don’t know		138	20.20%	75	16.90%	213	18.90%
Diabetes can damage my eyes	No		9	1.30%	9	2.00%	18	1.60%
	Yes	x	641	94.00%	423	95.10%	1,064	94.41%
	I don’t know		32	4.70%	13	2.90%	45	3.99%
Shaking and sweating are signs of high blood sugar	No	x	155	22.70%	151	33.90%	306	27.15%
	Yes		326	47.80%	210	47.20%	536	47.56%
	I don’t know		201	29.50%	84	18.90%	285	25.29%
Frequent urination and thirst are signs of low blood sugar	No	x	284	41.60%	170	38.20%	454	40.28%
	Yes		321	47.10%	242	54.40%	563	49.96%
	I don’t know		77	11.30%	33	7.40%	110	9.76%
Tight elastic hose or socks are not bad for diabetics	No	x	274	40.20%	237	53.30%	511	45.34%
	Yes		80	11.70%	55	12.40%	135	11.98%
	I don’t know		328	48.10%	153	34.40%	481	42.68%
A diabetic diet consists mostly of special foods	No		36	5.30%	38	8.50%	74	6.57%
	Yes	x	592	86.80%	387	87.00%	979	86.87%
	I don’t know		54	7.90%	20	4.50%	74	6.57%
Athletes are less prone to develop diabetes	No		79	11.60%	63	14.20%	142	12.60%
	Yes	x	465	68.20%	322	72.40%	787	69.83%
	I don’t know		138	20.20%	60	13.50%	198	17.57%
A fasting blood sugar range of 100–125 mg/dL indicates you have prediabetes	No		129	18.90%	135	30.30%	264	23.43%
	Yes	x	305	44.70%	208	46.70%	513	45.52%
	I don’t know		248	36.40%	102	22.90%	350	31.06%
Gestational diabetes increases future risk of type 2 diabetes?	No		46	6.70%	49	11.00%	95	8.43%
	Yes	x	364	53.40%	212	47.60%	576	51.11%
	I don’t know		272	39.90%	184	41.30%	456	40.46%

When stratifying the level of knowledge according to age group, in the age category of 40 years and younger (480 out of 1,127 participants), patients with T2DM (25 out of 480) were less likely to have poor knowledge than the control group (455 out of 480) (24% vs. 54%, p = 0.012). For participants above the age of 40 years (647 out of 1,127 participants), there was no significant difference in knowledge level between both groups (53% vs. 46%, p = 0.379). Knowledge (N = 1,127) was positively correlated with attitude (p < 0.001, r = 0.166; N = 445) and practice (p < 0.001, r = 0.169; N = 445).

Knowledge in the control group (N = 682) was significantly associated with higher educational attainment (p = 0.005), current employment (p = 0.025), and employment as a healthcare professional (p < 0.001). Multivariate analysis further confirmed that knowledge levels were significantly higher among healthcare professionals (p < 0.001, B = 0.232) and individuals with higher educational attainment (p = 0.007, B = 0.104) (Table 6).

**Table 6:** Multivariate analysis of factors affecting the level of knowledge about T2DM of the control group (N = 682). Dependent variable: knowledge. T2DM: type 2 diabetes mellitus

Model		Unstandardized coefficients		Standardized coefficients	T	P-value
		B	Standard error	Beta		
1	(Constant)	1.489	0.027		55.994	0
	Healthcare professional	0.522	0.074	0.262	7.094	0
2	(Constant)	1.271	0.085		14.91	0
	Healthcare professional	0.461	0.077	0.232	6.019	0
	Educational level	0.064	0.024	0.104	2.696	0.007

In the T2DM group (N = 445), knowledge was positively correlated with younger age (p = 0.048), employment status (p = 0.005), being a healthcare professional (p = 0.012), having a second-degree family member with diabetes (e.g., uncle, aunt, or grandparent) (p = 0.018), and awareness of HbA1c level (p = 0.005). Multivariate analysis further indicated that knowledge was significantly higher among younger patients (B = -0.160, p = 0.001), those currently employed (B = 0.107, p = 0.028), patients with higher educational attainment (B = 0.122, p = 0.012), and those aware of their HbA1c level (B = 0.180, p < 0.001) (Table 7).

**Table 7:** Multivariate analysis of factors affecting the level of knowledge about T2DM of the T2DM group (N = 445). Dependent variable: knowledge. T2DM: type 2 diabetes mellitus

Model	Unstandardized coefficients		Standardized coefficients	T	P-value
	B	Standard error	Beta		
(Constant)	1.343	0.096		13.93	0
Work	0.158	0.062	0.117	2.541	0.011
Do you have family members who are diabetic? (second degree: uncles, aunts, nephews, nieces, grandparents, grandchildren, half siblings, and double cousins)	0.127	0.062	0.093	2.026	0.043

Attitude assessment in T2DM patients

Of the 1,127 participants, only those with T2DM (445 participants) responded to the attitude-related questions. The attitude score about T2DM in the T2DM group was 101.49 ± 7.25 (145, 70%). More than half (51.9%, 231 out of 445 patients) exhibited a poor attitude toward T2DM, while only 3.6% (16 out of 445 patients) demonstrated a good attitude score (Figure 1, Panel b). Notably, the majority of participants did not believe that maintaining blood sugar levels close to normal could help prevent diabetes-related complications (68.1%, 303 of 445 patients). Additionally, most did not perceive diabetes as a serious disease (66.1%, 294 of 445 patients) and mistakenly believed that medication could be discontinued once diabetes was under control (68.1%, 303 of 445 patients) (Table 8).

**Table 8:** Distribution of participants according to their responses to attitude items about T2DM along with the respective mean score and standard deviation (N = 445). SD: standard deviation; T2DM: type 2 diabetes mellitus

Attitude items		Correct answer	Frequency	Percent	Mean	SD
Eating sweets occasionally is quite alright	Strongly disagree		39	8.8	3.38	1.12
	Disagree		74	16.6		
	Neutral		47	10.6		
	Agree		249	56.0		
	Strongly agree	x	36	8.1		
In general, I believe that there is not much use in trying to have good blood sugar control because the complications of diabetes will happen anyway	Strongly disagree	x	25	5.6	3.77	1.15
	Disagree		45	10.1		
	Neutral		72	16.2		
	Agree		168	37.8		
	Strongly agree		135	30.3		
In general, I believe that keeping the blood sugar close to normal can help to prevent the complications of diabetes	Strongly disagree		8	1.8	4.15	0.80
	Disagree		11	2.5		
	Neutral		32	7.2		
	Agree		250	56.2		
	Strongly agree	x	144	32.4		
In general, I believe that almost everyone with diabetes should do whatever it takes to keep their blood sugar close to normal	Strongly disagree		3	0.7	4.27	0.73
	Disagree		7	1.6		
	Neutral		34	7.6		
	Agree		223	50.1		
	Strongly agree	x	178	40.0		
In general, I believe that people who have diabetes will probably not benefit that much from tight control of their blood sugars	Strongly disagree	x	31	7.0	3.48	1.15
	Disagree		69	15.5		
	Neutral		80	18.0		
	Agree		187	42.0		
	Strongly agree		78	17.5		
Glycemic control is difficult to achieve	Strongly disagree		45	10.1	2.81	1.20
	Disagree	x	184	41.3		
	Neutral		73	16.4		
	Agree		95	21.3		
	Strongly agree		48	10.8		
Too frightened to eat fruits and sweets because of concerns about increased blood glucose	Strongly disagree		59	13.3	2.68	1.18
	Disagree	x	194	43.6		
	Neutral		54	12.1		
	Agree		105	23.6		
	Strongly agree		33	7.4		
Too frightened to take a meal (or reduce intake) because of concerns about increased postprandial glycaemia	Strongly disagree	x	50	11.2	2.73	1.18
	Disagree		193	43.4		
	Neutral		70	15.7		
	Agree		91	20.4		
	Strongly agree		41	9.2		
Even if I forget to take my medicines on some days, it is alright	Strongly disagree	x	19	4.3	3.72	1.22
	Disagree		82	18.4		
	Neutral		49	11.0		
	Agree		151	33.9		
	Strongly agree		144	32.4		
In general, I believe that people who do not need to take insulin to treat their diabetes have a pretty mild disease	Strongly disagree		30	6.7	3.32	1.18
	Disagree		95	21.3		
	Neutral		94	21.1		
	Agree		153	34.4		
	Strongly agree	x	73	16.4		
In general, I believe that people whose diabetes is treated by just a diet do not have to worry about getting many long-term complications	Strongly disagree	x	48	10.8	2.96	1.14
	Disagree		118	26.5		
	Neutral		118	26.5		
	Agree		127	28.5		
	Strongly agree		34	7.6		
In general, I believe that diabetes is a very serious disease	Strongly disagree		129	29.0	2.29	1.16
	Disagree		165	37.1		
	Neutral		58	13.0		
	Agree		77	17.3		
	Strongly agree	x	16	3.6		
In general, I believe that people who take diabetes oral pills should be as concerned about their blood sugar as people who take insulin injections	Strongly disagree		33	7.4	3.14	1.13
	Disagree		105	23.6		
	Neutral		124	27.9		
	Agree		132	29.7		
	Strongly agree	x	51	11.5		
Upon diabetes mellitus control, medicines can be stopped	Strongly disagree	x	23	5.2	3.71	1.15
	Disagree		60	13.5		
	Neutral		59	13.3		
	Agree		182	40.9		
	Strongly agree		121	27.2		
Lipid-lowering agents can help in T2DM?	Strongly disagree		1	0.2	3.97	0.82
	Disagree		15	3.4		
	Neutral		107	24.0		
	Agree		197	44.3		
	Strongly agree	x	125	28.1		
Switching to insulin indicates complications in T2DM?	Strongly disagree		8	1.8	3.49	0.94
	Disagree		49	11.0		
	Neutral		171	38.4		
	Agree		149	33.5		
	Strongly agree	x	68	15.3		
Hypoglycemia is more dangerous than hyperglycemia	Strongly disagree		16	3.6	3.71	1.10
	Disagree		45	10.1		
	Neutral		117	26.3		
	Agree		139	31.2		
	Strongly agree	x	128	28.8		
I should go for regular checkup as my doctor says, even if my sugars are under good control	Strongly disagree		7	1.6	4.18	0.83
	Disagree		18	4.0		
	Neutral		23	5.2		
	Agree		238	53.5		
	Strongly agree	x	159	35.7		
Even if I am not able to exercise as much as my doctor tells me to, it is alright because I get enough exercise while I am doing my daily activities	Strongly disagree	x	32	7.2	2.82	1.07
	Disagree		173	38.9		
	Neutral		111	24.9		
	Agree		99	22.2		
	Strongly agree		30	6.7		
In general, I believe that people with diabetes should learn a lot about the disease so that they can be in charge of their own diabetes care	Strongly disagree		5	1.1	4.25	0.77
	Disagree		12	2.7		
	Neutral		25	5.6		
	Agree		229	51.5		
	Strongly agree	x	174	39.1		
Is it important for a person with diabetes to control blood pressure?	Strongly disagree		7	1.6	4.04	0.87
	Disagree		22	4.9		
	Neutral		51	11.5		
	Agree		232	52.1		
	Strongly agree	x	133	29.9		
Do you think you should visit your physician regularly?	Strongly disagree		7	1.6	4.17	0.82
	Disagree		19	4.3		
	Neutral		20	4.5		
	Agree		245	55.1		
	Strongly agree	x	154	34.6		
Should a person with diabetes go for regular eye examination?	Strongly disagree		4	0.9	4.16	0.80
	Disagree		15	3.4		
	Neutral		41	9.2		
	Agree		229	51.5		
	Strongly agree	x	156	35.1		
In general, I believe that diabetes affects almost every part of a diabetic person’s life	Strongly disagree		26	5.8	3.58	1.16
	Disagree		77	17.3		
	Neutral		44	9.9		
	Agree		209	47.0		
	Strongly agree	x	89	20.0		
In general, I believe that the emotional effects of diabetes are pretty low	Strongly disagree	x	54	12.1	3.09	1.24
	Disagree		97	21.8		
	Neutral		108	24.3		
	Agree		125	28.1		
	Strongly agree		61	13.7		
In general, I believe that diabetes is hard because peoples with diabetes never get rid of it	Strongly disagree		17	3.8	3.72	1.10
	Disagree		65	14.6		
	Neutral		51	11.5		
	Agree		204	45.8		
	Strongly agree	x	108	24.3		
In general, I believe that having diabetes changes a person’s outlook on life	Strongly disagree		55	12.4	3.05	1.24
	Disagree		119	26.7		
	Neutral		68	15.3		
	Agree		155	34.8		
	Strongly agree	x	48	10.8		
In general, I believe that it is frustrating for people with diabetes to take care of their disease	Strongly disagree		74	16.6	2.74	1.23
	Disagree		157	35.3		
	Neutral		57	12.8		
	Agree		126	28.3		
	Strongly agree	x	31	7.0		
In general, I believe that support from family and friends is important in dealing with diabetes	Strongly disagree		8	1.8	4.09	0.89
	Disagree		23	5.2		
	Neutral		39	8.8		
	Agree		224	50.3		
	Strongly agree	x	151	33.9		

Attitude (N = 445) toward T2DM was positively correlated with knowledge about T2DM (p < 0.001, r = 0.166, N = 1,127) and practice (r = 0.010) but without reaching statistical significance with practice (p = 0.841). Attitude also positively correlated with the marital status (single or divorced) (p = 0.015) and the city of origin (being from Beirut governorate; p = 0.016). In addition, having a second-degree family member with T2DM (p = 0.039) and being aware of their HbA1c level (p = 0.003) were positively correlated. Multivariate analysis showed that higher attitude was associated with higher knowledge about T2DM (B = 0.114, p = 0.016), patients who were not from Mount Lebanon (B = -0.106, p = 0.024), and those with a family member with T2DM (B = 0.103, p = 0.030) (Table 9).

**Table 9:** Multivariate analysis of factors affecting the level of attitude toward T2DM in the T2DM group (N = 445). Dependent variable: attitude. T2DM: type 2 diabetes mellitus

Model	Unstandardized coefficients		Standardized coefficients	T	P-value
	B	Standard error	Beta		
(Constant)	1.127	0.151		7.478	0
Knowledge	0.013	0.005	0.114	2.417	0.016
Mount Lebanon	-0.13	0.058	-0.106	-2.266	0.024
Do you have family members who are diabetic? (second degree: uncles, aunts, nephews, nieces, grandparents, grandchildren, half siblings, and double cousins)	0.119	0.055	0.103	2.181	0.03

Practice assessment in T2DM patients

Of the 1,127 participants, only those with T2DM (445 participants) responded to the practice-related questions. The average practice score in this group was 10.49 ± 2.66 out of 16 (65.58%). Notably, 86.7% of participants exhibited a “Limited” practice level toward T2DM, with 60.7% classified as having poor practice and 26.1% as fair practice scores. In contrast, only 13.3% (59 of 445) demonstrated an “Adequate” practice level, achieving good or excellent practice scores (Figure 1, Panel c). For instance, while the majority of patients with T2DM adhered to their prescribed medication regimen (95.1%, 423 of 445) and took adequate precautions while cutting their nails (82%, 365 of 445), fewer engaged in other essential self-care practices. Only 29.7% (132 of 445) followed a regular exercise routine, while a significant proportion were smokers (58.7%, 261 of 445) and had experienced hypoglycemia due to irregular lifestyle choices (60.7%, 270 of 445) (Table 10).

**Table 10:** Distribution of participants according to their responses to practice items about T2DM (N = 445). T2DM: type 2 diabetes mellitus

Practice items		Correct answer	Frequency	Percent
Do you take medicines for diabetes as advised by the physician?	No		22	4.9
	Yes	x	423	95.1
Do you take regular exercise?	No		313	70.3
	Yes	x	132	29.7
Do you smoke (nargileh, vaping, electronic cigarette)?	No	x	184	41.3
	Yes		261	58.7
Do you have any exposure to passive smoking (do you sit near smoking peoples who smoke cigarettes or nargileh)?	No	x	297	66.7
	Yes		148	33.3
Is your diabetes under control at present?	No		65	14.6
	Yes	x	380	85.4
Do you follow low sugar diet?	No		164	36.9
	Yes	x	281	63.1
Do you control your weight?	No		169	38.0
	Yes	x	276	62.0
Have you experienced hypoglycemia due to irregular life style choices?	No	x	175	39.3
	Yes		270	60.7
Have you experienced between-meal hypoglycemia, bedtime hypoglycemia, or nocturnal hypoglycemia?	No	x	155	34.8
	Yes		290	65.2
Do you regularly monitor your glucose level during the day?	No		208	46.7
	Yes	x	237	53.3
Do you regularly monitor your blood pressure?	No		174	39.1
	Yes	x	271	60.9
Do you take adequate care while cutting nails?	No		80	18.0
	Yes	x	365	82.0
If you forget to take your diabetes medications, do you know how to act in this case?	No		160	36.0
	Yes	x	285	64.0
Do you ask your physician or the pharmacist for recommendations or advice concerning your diabetes disease?	No		104	23.4
	Yes	x	341	76.6
Do you go for regular follow-up to your physician?	No		149	33.5
	Yes	x	296	66.5
Have you been hospitalized in the past 30 days for complications of diabetes?	No	x	32	7.2
	Yes		413	92.8

Practice (N = 445) toward T2DM was positively correlated with knowledge about T2DM (p < 0.001, r = 0.169, N = 1,127) and practice (r = 0.010) but without reaching statistical significance with practice (p = 0.841, N = 445). Practice level toward T2DM was positively correlated with educational attainment (p = 0.045) and smoking status (p < 0.001), with smokers exhibiting the highest prevalence of poor practice (80.2%, 146 of 182 patients) compared to non-smokers (47.2%, 101 of 214 patients) and ex-smokers (46.9%, 23 of 49 patients). Additionally, practice level was negatively associated with obesity (p = 0.016), as obese individuals had a higher prevalence of poor practice (62.7%, 101 of 161 patients) compared to non-obese individuals (59.5%, 169 of 284 patients). Multivariate analysis revealed that good practice toward T2DM was more prevalent among non-smokers (B = -0.314, p < 0.001), individuals with higher knowledge about T2DM (B = 0.147, p = 0.001), those with higher educational attainment (B = 0.097, p = 0.032), and non-obese individuals (B = -0.126, p = 0.005) (Table 11).

**Table 11:** Multivariate analysis of factors affecting the level of practice toward T2DM in the T2DM group (N = 445). Dependent variable: practice. T2DM: type 2 diabetes mellitus

Model	Unstandardized coefficients		Standardized coefficients	T	P-value
	B	Standard error	Beta		
(Constant)	1.719	0.043		39.684	0
Do you smoke?	-0.455	0.068	-0.304	-6.717	0
(Constant)	1.422	0.092		15.528	0
Do you smoke?	-0.456	0.067	-0.304	-6.82	0
Knowledge	0.18	0.049	0.163	3.656	0
(Constant)	1.687	0.131		12.856	0
Do you smoke?	-0.473	0.067	-0.316	-7.105	0
Knowledge	0.181	0.049	0.164	3.706	0
Obesity	-0.191	0.068	-0.125	-2.8	0.005
(Constant)	1.499	0.157		9.528	0
Do you smoke?	-0.47	0.066	-0.314	-7.094	0
Knowledge	0.161	0.049	0.147	3.27	0.001
Obesity	-0.192	0.068	-0.126	-2.837	0.005
Educational level	0.088	0.041	0.097	2.155	0.032

## Discussion

**Figure 2:**
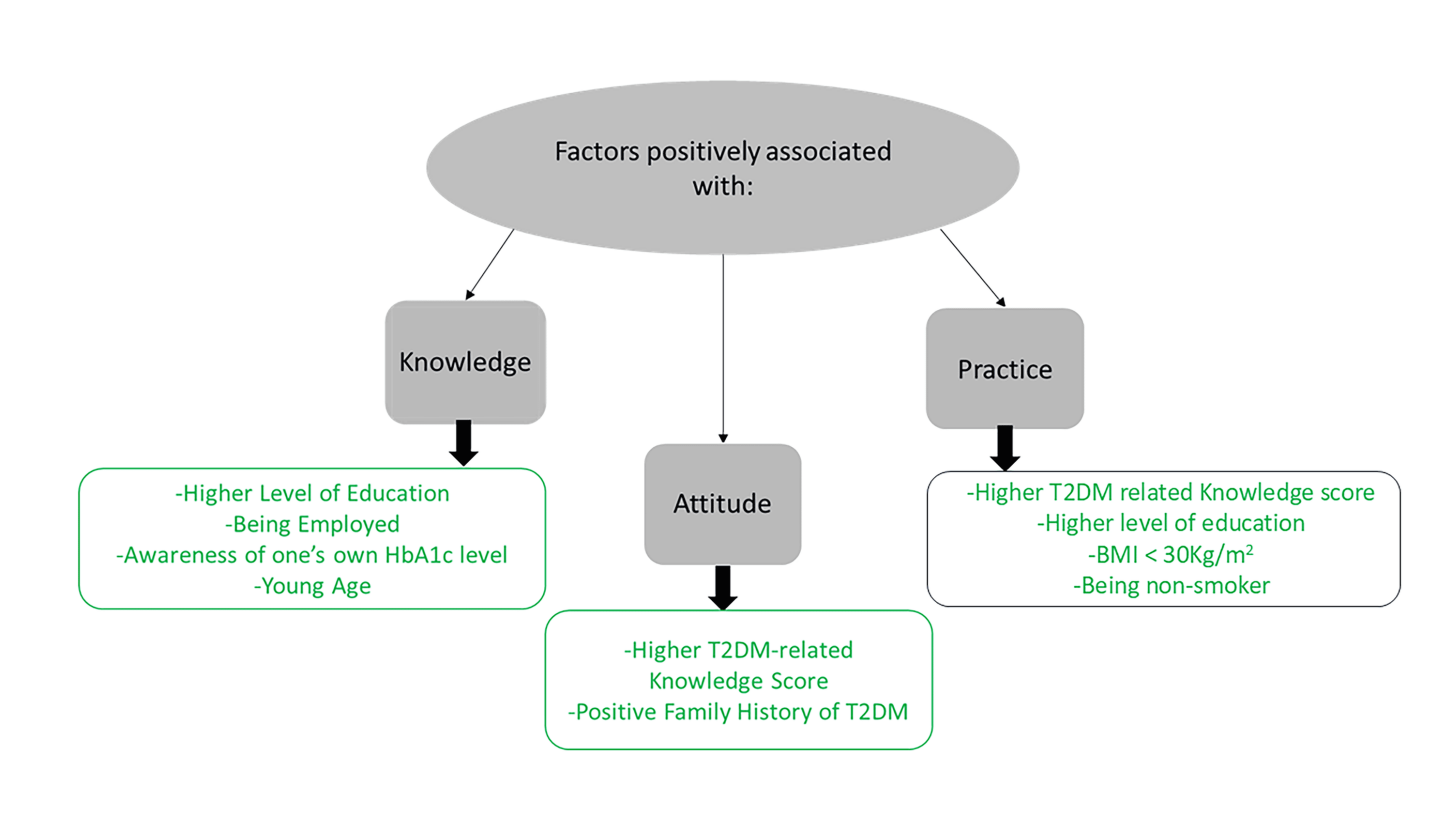
Factors associated with higher T2DM-related KAP scores. T2DM: type 2 diabetes mellitus; BMI: body mass index; KAP: knowledge, attitude, and practice

Assessing the KAP levels regarding T2DM is crucial, particularly considering its increasing prevalence within the Lebanese population [6]. Following the 2018 Lebanese study conducted by Karaoui et al., it was reported that there were generally low levels of knowledge and practice regarding T2DM [9]. Our study also evaluated another important aspect, attitude, which was found to be unsatisfactory. We demonstrated a limited level of awareness regarding T2DM, as evidenced by low KAP scores in the Lebanese population. Several factors were also shown to be associated with KAP levels, as summarized in Figure 2.

Knowledge

A considerable proportion (91%) of participants, either healthy or diabetic, displayed “Limited” knowledge regarding T2DM, with half of the Lebanese population displaying a poor level of knowledge. Interestingly, studies conducted in the MENA region yielded conflicting results. For instance, a multicentric Iranian study demonstrated that approximately 61% of participants had good knowledge [21]. In contrast, recent studies conducted in Saudi Arabia [10] and Qatar [11] indicated a low level of diabetes-related knowledge among most participants (62.4% and 69%, respectively), consistent with other reports from China [22] (63% of participants). A possible explanation for the low knowledge score among the Lebanese population could be the socioeconomic status of the country at the time of data collection. The concurrent challenges of the COVID-19 pandemic and the economic crisis in Lebanon likely had profound negative impacts on the psychological well-being and the allocation of resources, including attention to health-related matters, across the entire population. Given that enhanced knowledge about T2DM can contribute to better disease management [9], especially considering the observed low adherence to medications among Lebanese diabetic individuals [23], the importance of targeted interventions to improve this knowledge cannot be undermined. Interestingly, similar trends concerning an unsatisfactory score have emerged in several recent KAP studies about other chronic diseases within the Lebanese population [19,20]. This study demonstrated a positive correlation between awareness of one’s HbA1c levels and knowledge about T2DM. This finding is consistent with the study by Karaoui et al., which linked greater T2DM knowledge to improved adherence to appropriate self-care practices [9]. Additionally, research by Mroueh et al. highlighted an association between better glycemic control, as indicated by HbA1c levels, and increased adherence to antidiabetic medications [23]. Collectively, these findings reinforce a comprehensive relationship between awareness of HbA1c levels, enhanced T2DM knowledge, and the adoption of more effective disease management practices.

In the Lebanese population, the limited level of knowledge related to T2DM may be due to low health literacy, in general, or, from the physician’s perspective, an oversimplified explanation of several aspects of diabetes to the patients to limit confusion, thus potentially omitting significant information such as HbA1c’s implication in monitoring the progression of the disease. Furthermore, HbA1c levels are significantly associated with the quality of life of the diabetic patient [7] and the possibility of developing diabetes-related distress [24]. Hence, it is imperative to prioritize attention toward understanding HbA1c levels, aiming to elevate knowledge in this area, with the potential outcome of enhancing adherence to best practices in managing T2DM.

Notably, age was found to be inversely associated with T2DM knowledge, consistent with recent findings from China [25]. In contrast, Karaoui et al. reported no significant relationship between age and T2DM knowledge in the Lebanese population, instead suggesting that younger individuals’ better understanding of the disease was primarily linked to higher practice scores [9]. This discrepancy may be attributed to the relatively small sample size (207 participants) in the study by Karaoui et al. [9].

Similar to various recent studies [9,25,26], a higher level of education was associated with better T2DM knowledge. Health literacy has been highlighted by a recent Lebanese study [27] as a crucial factor in reducing and preventing diabetes-related complications, thus stressing the necessity of better comprehensive educational programs. An Iranian study found an inverse relationship between the time since graduation and the corresponding knowledge [21], an aspect that is yet to be assessed within the Lebanese population. Hence, this interesting dimension, if confirmed, would stress the importance of the long-term continuity of education in acquiring or maintaining additional knowledge regarding diabetes. Our study also indicated better knowledge among working participants in agreement with other recent studies [25], yet failed to find a significant association with household income. This could be due to occasional awareness campaigns organized within certain occupational environments, and easier accessibility to physicians and healthcare platforms in certain types of occupations, regardless of the financial aspect. In addition, Lebanon ranks among the world’s most remittance-dependent countries in the world [28], which could explain the discordance between the “working” and “household income” categories. No significant gender-based association with T2DM knowledge level was demonstrated, which confirms previous results by Karaoui et al [9].

Attitude

The majority of participants exhibited a limited attitude toward T2DM (96%), with half of those with T2DM displaying a poor attitude score. Similar findings have been reported in various countries, including China [28], where 62% of participants demonstrated a poor attitude. In contrast, recent studies in Qatar [11] and South Africa [26] reported more favorable attitudes toward T2DM, with 87% and 50.4% of participants, respectively, exhibiting adequate attitude levels.

This widespread poor attitude toward T2DM is likely attributed to equally low levels of knowledge. As emphasized by Mousavi and Shojaei, a significant positive correlation exists between knowledge and attitude levels [29]. However, a recent study in China [25] did not establish such a relationship but suggested that better knowledge contributes to improved diabetes management practices.

Interestingly, our findings, in agreement with Alenbalu et al. [26], indicate a significant association between knowledge level and both attitude and practice. Additionally, our study demonstrated that a positive family history of diabetes is linked to a more favorable attitude toward the disease. This relationship can be explained by a heightened sense of care for affected family members and an increased personal awareness of the risk of developing diabetes.

Practice

Around 61% of our respondents exhibited poor practice toward T2DM, culminating in a limited practice score within most of the Lebanese diabetic population (87%). Primarily, low levels of physical activity were recorded, yet not to the extent previously described by Karaoui et al. [9] (70% vs. 84% of participants, respectively). Contrary to our results, Mroueh et al. indicated a low level of adherence to oral antidiabetic medication [23]. As our study was conducted during the COVID-19 pandemic, this discrepancy could be explained by the heightened levels of health-related fear and precautions undertaken, especially in diabetic patients, who are already subject to a higher level of morbidity and mortality at baseline. Another possible explanation could reside in the inability to assess the actual consistency and regularity of compliance with the prescribed medication through our close-ended question on the topic. In accordance with Karaoui et al.’s results [9], our study indicated a significant positive association between the level of education and T2DM-related practice, as replicated by various studies [25,26]. Concomitantly, a better knowledge score was also associated with better overall practice regarding diabetes, reiterating previous results from Lebanon [9], Iran [29], and China [25]. A recent study by Abboud et al. indicated healthier eating habits in Lebanese diabetic patients with higher dietary knowledge [30]. This also serves to support our results regarding the negative association between obesity and practice score.

Study limitations

This study presents several limitations. No causality could be confirmed between potential factors and KAP scores due to the cross-sectional nature of this study. In addition, although the majority (96%) of the interviews were conducted via phone calls or even face-to-face, the inevitable presence of a certain degree of recall, misreporting, and selection biases cannot be denied, This survey had a restricted capacity to reach the illiterate or underprivileged population lacking technological practicalities (phones, internet), leading to a sampling bias due to overrepresentation of the literate and educated subsets of the population. It is also crucial to acknowledge a form of social desirability bias as some participants might have provided socially desirable responses rather than their actual opinions, along with another potential bias, the central tendency bias associated with a tendency to avoid “extremes” and instead choose neutral responses when answering attitude-related questions utilizing the Likert scale.

While we used Google Forms to structure and standardize our work, we conducted interviews to ensure inclusivity (sampling bias) and accuracy of data collection (reporting bias). Many participants, specifically the elderly, might face difficulty navigating Google Forms independently. In addition, interviews allowed us to reach out to participants with limited internet access and digital literacy. Conducting interviews helped minimize reporting bias that could arise from misunderstanding questions. We also considered volunteer bias by interviewing participants in both healthcare and community settings rather than relying solely on self-selected online participants.

Study perspectives and recommendations

According to our findings, the instauration of adequate public health strategies that target the entire Lebanese population, with a particular focus on rural areas, is crucial for better T2DM-related KAP levels. Future studies should assess T2DM-related attitudes and practices in non-diabetics to identify health gaps, their influence on T2DM patients, and their own risk of developing diabetes. This research would help design targeted public health interventions. Our study also indicates that additional awareness regarding certain reversible lifestyle parameters, including obesity and smoking, should be raised to potentially improve practice among diabetic patients. Importantly, the development of efficient educational programs adapted to various age, occupational, and educational categories should be prioritized, mainly through audiovisual platforms, including videos, pamphlets, and mobile applications. 

Importantly, future research should focus on the effect of educational workshops and interventional programs on the level of awareness regarding diabetes. Particularly, studies should measure the percent change in KAP. In addition, conducting longitudinal cohort and mixed-method analysis will help better understand factors influencing T2DM individuals’ awareness, document behavioral changes over time, and ensure up-to-date tailored interventions.

Given the high regard for healthcare workers’ advice and recommendations in the Lebanese culture, reassessing the way healthcare professionals engage with diabetic patients and subsequently reformulating the approach to patient education and communication, could significantly impact how individuals manage their diabetes. In addition, further investigations targeting the main gaps mentioned within this study, particularly concerning knowledge and practice, should be undertaken, including a deeper evaluation of the psychological impact of certain practice-related factors on the quality of life of diabetic patients.

## Conclusions

This study revealed that the population demonstrated low levels of KAP regarding T2DM. These findings highlight the pressing need for additional efforts directed toward enhancing overall awareness and promoting appropriate practices related to T2DM within the Lebanese population to address the current situation. This highlights the need for tailored awareness campaigns conducted by the Ministry of Public Health, universities, and non-governmental organizations that target gaps in knowledge, wrong perceptions, poor attitudes, and flawed practices to effectively improve T2DM awareness and management.

## Appendices

Study questionnaire

**Table 12:** Study questionnaire: English and Arabic versions.

English version
Knowledge, Attitude, and Practice (KAP) Toward Type 2 Diabetes Mellitus Among Lebanese Residents. Contribute to this scientific research whether you suffer or not from diabetes. A research team from the Faculty of Medical Sciences at the Lebanese University is carrying out a study on the level of knowledge about diabetes among the Lebanese people (whether you do or do not suffer from diabetes), in addition to its impact on patients’ lives and behavior. If you have any questions, you can contact us at kapdiabetes@gmail.com. We hope that you will answer the following questions honestly and accurately, and we confirm that the information that you will provide will only be used for purely scientific reasons. You have the right to leave the study anytime! Your answers will be completely anonymous.
1. The information you provide here will be used in the above study and not for any other purpose. Do you agree to participate? Yes No Skip to Section 13 (Thanks for your participation!)
Before you start: 2. You are a Lebanese citizen, residing in Lebanon, older than 18 years old *Mark only one oval. Yes No Skip to Section 13 (Thanks for your participation!)
General information
3. What is your gender? Female Male
4. How old are you
5. Please select your marital status? Single Married Divorced Widower
6. Please specify the governorate you live in: Beirut Mount Lebanon North Lebanon South Lebanon Bekaa Akkar Nabatieh Baalbek-Hermel
7. Please select the highest level of you education you have completed. And in case you are still completing your studies, choose the degree you are currently enroled (the one you are studying): I did not go to school Elementary school Baccalaurate Bachelor degree Master degree Doctorate/PhD Medical degree
8. What type of work do you do? I’m a healthcare professional I work but I’m not a healthcare professional I don’t work
9. Do you have family members who are diabetic? First degree (father, mother, full siblings, child) Yes No Second degree (uncles, aunts, nephews, nieces, grandparents, grandparents, grandchildren, half siblings, and double cousins) Yes No
10. Do you smoke? Yes No Ex-smoker <15 Ex-smoker >15
11. What is your weight? In kilograms, kg
12. What is your height? In centimeters, cm
13. Do you drink alcohol? Yes, regularly Yes, occasionally No
14. What chronic diseases do you suffer from? Hypertension Yes No Cardiovascular Yes No diseases other than hypertension (e.g., heart failure, coronary artery disease) Yes No Cancer Yes No Lung Disease Yes No Chronic Lung Disease (e.g., Chronic obstructive pulmonary disease, asthma) Yes No Renal failure Yes No Other diseases Yes No
Knowledge: Please don’t get assistance neither from the internet nor from a book. Please just respond according to “your” knowledge!
15. What are the risk factors you think that contribute to diabetes? Obesity Yes No I don’t know Decreased physical activity Yes No I don’t know Family history of diabetes Yes No I don’t know Mental stress Yes No I don’t know Consuming more sweets Yes No I don’t know Smoking Yes No I don’t know Being skinny Yes No I don’t know Doing physical exercise Yes No I don’t know Others Yes No I don’t know
16. Do you know how to measure diabetes (glucose (sugar))? Yes No
17. Does diabetes cause complications in other organs? Yes No I don’t know
18. Can diabetes be prevented? Yes No I don’t know
19. What are the tests done to diagnose diabetes (to find out if a person is diabetic)? Blood tests (glycemia, HbA1c) Yes No I don’t know Urine tests Yes No I don’t know Any other Yes No I don’t know
20. How can you keep diabetes under control? Medication Yes No I don’t know Diet Yes No I don’t know Exercise Yes No I don’t know Weight reduction Yes No I don’t know Going for regular check-up Yes No I don’t know Any other Yes No I don’t know
21. Eating too much sugar and other sweet foods is a cause of diabetes. Yes No Don’t know
22. The usual cause of diabetes is lack of effective insulin in the body. Yes No Don’t know
23. Diabetes is caused by failure of the kidneys to keep sugar out of the urine. Yes No Don’t know
24. Kidneys produce insulin. Yes No Don’t know
25. In untreated diabetes, the amount of sugar in the blood usually increases. Yes No Don’t know
26. If I am diabetic, my children have a higher chance of being diabetic. Yes No Don’t know
27. Diabetes can be cured. Yes No Don’t know
28. A fasting blood sugar level of 210 mg/dL is too high. Yes No Don’t know
29. Regular exercises will increase the need for insulin or other diabetic medication. Yes No Don’t know
30. There are two main types of diabetes: Type 1 (insulin dependent) and Type 2 (non-insulin dependent) Yes No Don’t know
31. Medication is more important than diet and exercise to control my diabetes. Yes No Don’t know
32. Diabetes often causes poor circulation. Yes No Don’t know
33. Wounds heal more slowly, in diabetic patients. Yes No Don’t know
34. Diabetics should take extra care when cutting their toe nails. Yes No Don’t know
35. A person with diabetes should cleanse a cut with iodine and alcohol. Yes No Don’t know
36. The way I prepare my food is as important as the foods I eat. Yes No Don’t know
37. Diabetes can damage my kidneys Yes No Don’t know
38. Diabetes can damage my eyes. Yes No Don’t know
39. Shaking and sweating are signs of high blood sugar. Yes No Don’t know
40. Frequent urination and thirst are signs of low blood sugar. Yes No Don’t know
41. Tight elastic hose or socks are good for diabetics. Yes No Don’t know
42. A diabetic diet consists mostly of special foods. special foods examples: vegetables, fruits, grains, non-fat or low fat dairy, protein sources from: fish, chicken or turkey without the skin, lean meat. Yes No Don’t know
43. Athletes are less prone to develop diabetes. Yes No Don’t know
44. A fasting blood sugar range of 100 to 125 mg/dL indicates you have prediabetes. Yes No Don’t know
45. Gestational diabetes increases future risk of type 2 diabetes? Yes No Don’t know
Diabetes
46. Do you suffer from diabetes mellitus? Yes No (Thanks for your participation!)
General information for diabetic patients
47. Which type of diabetes do you suffer from? Type 1 (formerly known as juvenile diabetes). (Thanks for your participation!) Type 2 (formerly known as adult-onset diabetes)
48. For how many years have you been diabetic? Please state the number of years since you were diagnosed with the disease, not the date of diagnosis (e.g.,, if you were diagnosed with diabetes 8 years ago, just write: 8)
49. Do you take insulin for diabetes? Yes No
50. Do you know what your HbA1c level is? * known in Arabic as السكر مخزون Yes No Skip to question 52
51. What’s your HbA1c level? Less than 5.7% Between 5.7% and 6.4% Between 6.4 and 7% Between 7 and 8% Equal or More than 8%
Attitude
52. Eating sweets occasionally is quite alright. Strongly Disagree Disagree Neutral Agree Strongly Agree
53. Even if I forget to take my medicines on some days, it is alright. Strongly Disagree Disagree Neutral Agree Strongly Agree
54. I should go for regular checkup as my doctor says, even if my blood sugar is under good control. Strongly Disagree Disagree Neutral Agree Strongly Agree
55. Even if I am not able to exercise as much as my doctor tells me to, it is alright because I get enough exercise while I am doing my daily activities. Strongly Disagree Disagree Neutral Agree Strongly Agree
56. In general, I believe that people who do not need to take insulin to treat their diabetes have a pretty mild disease. Strongly Disagree Disagree Neutral Agree Strongly Agree
57. In general, I believe that there is not much use in trying to have good blood sugar control because the complications of diabetes will happen anyway. Strongly Disagree Disagree Neutral Agree Strongly Agree
58. In general, I believe that diabetes affects almost every part of a diabetic person’s life. Strongly Disagree Disagree Neutral Agree Strongly Agree
59. In general, I believe that diabetes affects almost every part of a diabetic person’s life. Strongly Disagree Disagree Neutral Agree Strongly Agree
60. In general, I believe that keeping the blood sugar close to normal can help to prevent the complications of diabetes. Strongly Disagree Disagree Neutral Agree Strongly Agree
61. In general, I believe that people whose diabetes is treated by just a diet do not have to worry about getting many long-term complications. Strongly disagree Disagree Neutral Agree Strongly Agree
62. In general, I believe that almost everyone with diabetes should do whatever it takes to keep their blood sugar close to normal. Strongly Disagree Disagree Neutral Agree Strongly Agree
63. In general, I believe that the emotional effects of diabetes are pretty low. Strongly Disagree Disagree Neutral Agree Strongly Agree
64. In general, I believe that diabetes is hard because peoples with diabetes never get rid of it. Strongly Disagree Disagree Neutral Agree Strongly Agree
65. In general, I believe that diabetes is a very serious disease. Strongly disagree Disagree Neutral Agree Strongly Agree
66. In general, I believe that having diabetes changes a person’s outlook on life. Strongly disagree Disagree Neutral Agree Strongly Agree
67. In general, I believe that people who have diabetes will probably not benefit that much from tight control of their blood sugars. Strongly Disagree Disagree Neutral Agree Strongly Agree
68. In general, I believe that people with diabetes should learn a lot about the disease so that they can be in charge of their own diabetes care. Strongly Disagree Disagree Neutral Agree Strongly Agree
69. In general, I believe that it is frustrating for people with diabetes to take care of their disease. Strongly Disagree Disagree Neutral Agree Strongly Agree
70. In general, I believe that people who take diabetes oral pills should be as concerned about their blood sugar as people who take insulin injections. Strongly Disagree Disagree Neutral Agree Strongly Agree
71. In general, I believe that support from family and friends is important in dealing with diabetes. Strongly Disagree Disagree Neutral Agree Strongly Agree
72. Is it important for a person with diabetes to control blood pressure? Strongly Disagree Disagree Neutral Agree Strongly Agree
73. Do you think you should visit your physician regularly? Strongly Disagree Disagree Neutral Agree Strongly Agree
74. Upon diabetes mellitus control, medicines can be stopped Strongly Disagree Disagree Neutral Agree Strongly Agree
75. Should a person with diabetes go for regular eye examination? Strongly Disagree Disagree Neutral Agree Strongly Agree
76. Lipid-lowering agents can help in type 2 diabetes mellitus? Strongly Disagree Disagree Neutral Agree Strongly Agree
77. Switching to insulin indicates complications in type 2 diabetes mellitus? oval. Strongly Disagree Disagree Neutral Agree Strongly Agree
78. Hypoglycemia is more dangerous than hyperglycemia? Hypoglycemia is a condition in which your blood sugar (glucose) level is lower than normal, hyperglycemia is a condition in which your blood sugar (glucose) level is higher than normal. Strongly Disagree Disagree Neutral Agree Strongly Agree
79. Glycemic control is difficult to achieve? * Strongly Disagree Disagree Neutral Agree Strongly Agree
80. Too frightened to eat fruits and sweets because of concerns about increased blood glucose. Strongly Disagree Disagree Neutral Agree Strongly Agree
81. Too frightened to take a meal (or reduce intake) because of concerns about increased postprandial glycemia. Postprandial means after eating a meal. Strongly Disagree Disagree Neutral Agree Strongly Agree
Practice
82. Do you take medicines for diabetes as advised by the physician? Yes No
83. Do you do regular exercise? Yes No
84. Do you smoke (nargileh, vaping, electronic cigarette)? Yes No
85. Do you have any exposure to passive smoking (do you sit near smoking people who smoke cigarettes or nargileh)? Yes No
86. Is your diabetes under control at present? Under control: e.g., keeping your blood sugar levels within healthy limits. Yes No Don’t know
87. How often do you visit your physician? Daily Weekly Monthly Within 2–3 Months More than 3 months
88. Do you follow low sugar diet? Yes No
89. Do you control your weight? Yes No
90. Have you experienced hypoglycemia due to irregular life style choices? Hypoglycemia is a condition in which your blood sugar (glucose) level is lower than normal. Yes No
91. Have you experienced between-meal hypoglycemia, bedtime hypoglycemia, or nocturnal hypoglycemia? Hypoglycemia is a condition in which your blood sugar (glucose) level is lower than normal. Yes No
92. Do you take regularly your glucose level during the day? Yes No
93. Do you regularly monitor your blood pressure? Yes No
94. How many times did you visit an eye doctor during the last 5 years? 0 1 2 3 4 5 >5
95. Do you take adequate care while cutting nails? Yes No
96. If you forget to take your diabetes medications, do you know how to act in this case? Yes No
97. Do you ask your physician or the pharmacist for recommendations or advices concerning your diabetes disease? Yes No
98. Do you go for regular follow-up to your physician? Yes Skip to question 99 No
99. Why do you not go for regular follow up to your physician? Check all that apply. Cannot afford No family supports Do not think it is important Did not find time Checking sugar levels with glucometer at home is sufficient Did not know that regular follow up is necessary Others
100. Have you been hospitalized in the past 30 days for complications of diabetes? Yes No
المعرفة السلوك والممارسة تجاه مرض B- Arabic Version: السكري من النوع الثاني بين السكان اللبنانيين المقيمين
ساهم بهذا البحث العلمي سواء "كنت تعاني" او "ال تعاني" من داء السكري. يقوم فريق بحثي من كلية العلوم الطبية في الجامعة اللبنانية، بدراسٍة حول مستوى المعرفة حول مرض السكري لدى الشعب اللبناني (سواء كنت تعاني من مرض السكري أم لا)، اضافة الى تأثيره على حياة المرضى وسلوكهم. اذا كان لديك أي أسئلة الرجاء التواصل معنا عبر: kapdiabetes@gmail.com نرجو منكم اإلجابة بصدق ودقة عن األسئلة التالية، ونتعهد اليكم ان المعلومات التي ستعطونها لن ُتستعمل اال ألسباب علمية بحتة. لديك الحق في ترك الدراسة في أي وقت! ستكون إجاباتك مجهولة تماًما.
سيتم استخدام المعلومات التي تقدمها هنا في الدراسة أعاله وليس ألي غرض آخر. هل توافق على المشاركة؟ نعم لا --> شكرا لمشاركتك. الذهاب الى section 13
انت لبناني, مقيم في لبنان, وعمرك أكثر من 18 سنة نعم لا --> شكرا لمشاركتك. الذهاب الى section 13
معلومات عامة
ما هو جنسك؟ أنثى ذكر
كم عمرك ؟
يرجى تحديد حالتك االجتماعية أعزب متزوج/ة مطلق/ة أرمل/ة
يرجى تحديد المحافظة التي تنتمي(ن) اليها بيروت جبل لبنان شمال لبنان جنوب لبنان البقاع عكار النبطية بعلبك الهرمل
يرجى تحديد أعلى مستوى من التعليم أكملت. و في حال ما زلت تكمل دراستك اختر الشهادة المسجل بها حالي ًا: (أي التي تدرسها) لم أدخل المدرسة المدرسة االبتدائية شهادة البكالوريا درجة البكالريوس درجة الماجستير درجة الدكتوراة شهادة طب
ما نوع العمل الذي تقوم به؟ أنا متخصص في الرعاية الصحية أنا أعمل ولكني لست متخصًصا في الرعاية الصحية لا أعمل
هل لديك أفراد من عائلتك مصابون بالسكري؟ الدرجة الأولى: أب ، أم ، إخوة أشقاء ، ابناء نعم كلا الدرجة الثانية: الأعمام ، العمات ، أبناء اإلخوة ، بنات االخوة، األجداد ، األحفاد ، نصف األشقاء وأبناء العم نعم كلا
هل تدخن؟ نعم لا (مدخن سابق ) توقفت عن التدخين منذ "أقل" من 15 سنة (مدخن سابق )توقفت عن التدخين منذ "أكثر" من 15 سنة
كم وزنك؟
ما طولك؟
هل تشرب الكحول؟ نعم، بانتظام نعم ، من حين لآخر كلا
ما هي األمراض المزمنة التي تعاني منها؟ ارتفاع ضغط الدم نعم كلا أمراض القلب والأوعية الدموية بخلاف ارتفاع ضغط الدم )مثال: قصور القلب ، ومرض الشريان التاجي) سرطان نعم كلا أمراض الرئة المزمنة مثل: مرض االنسداد الرئوي المزمن ، الربو نعم كلا الفشل الكلوي نعم كلا أمراض أخرى نعم كلا
المعرفة: يرجى عدم الحصول على مساعدة من اإلنترنت وال من أي كتابز يرجى فقط الرد وفقا لمعرفتك الخاصة بك
ما هي عوامل الخطر التي تعتقد أنها تساهم في اإلصابة بمرض السكري السمنة نعم لا لا أعرف قلة النشاط البدني نعم لا لا أعرف تاريخ عائلي للاصابة بمرض السكري نعم لا لا أعرف الضغط النفسي نعم لا لا أعرف تناول المزيد من الحلويات نعم لا لا أعرف التدخين نعم لا لا أعرف نحيف البدن نعم لا لا أعرف القيام بالتمارين الرياضية نعم لا لا أعرف عوامل اخرى نعم لا لا أعرف
هل تعرف كيف تقيس نسبة السكري (الجلوكوز السكر) ؟ نعم لا
هل يمكن أن يسبب مرض السكري مضاعفات في أعضاء أخرى؟ نعم لا لا أعرف
هل يمكن تجنب مرض السكري؟ نعم لا لا أعرف
ما هي الفحوصات التي يتم إجراؤها لتشخيص مرض السكري (لمعرفة ما إذا كان الشخص مصابا بمرض السكر)؟ تحاليل الدم (سكر الدم، سكر المخزون) نعم لا لا أعرف فحوصات البول نعم لا لا أعرف تحاليل اخرى نعم لا لا أعرف
كيف يمكنك السيطرة على مرض السكري؟ الدواء نعم لا لا أعرف اتباع حمية نعم لا لا أعرف تمرين إنقاص الوزن نعم لا لا أعرف الفحص المنتظم نعم لا لا أعرف إجراءات اخرى نعم لا لا أعرف
اإلكثار من تناول السكر واألطعمة الحلوة األخرى يسبب مرض السكري نعم لا لا أعرف
السبب المعتاد لمرض السكري هو نقص األنسولين الفعال في الجسم نعم لا لا أعرف
مرض السكري ناتج عن فشل الكلى في إبقاء السكر خارج البول نعم لا لا أعرف
نتج الكلى األنسولين نعم لا لا أعرف
تزداد عادة كمية السكر في الدم في حال عدم عالج مرض السكري نعم لا لا أعرف
إذا كنت مصاًبا بالسكري فإن أطفالي عرضة اكثر لإلصابة بمرض السكري نعم لا لا أعرف
بالامكان الشفاء من مرض السكري نعم لا لا أعرف
يعد مستوى السكر 210 ملغ / ديسيلترفي الدم الصائم مرتفع جدًا نعم لا لا أعرف
تزيد التمارين المنتظمة من الحاجة إلى األنسولين أو أدوية السكري الاخرى نعم لا لا أعرف
هناك نوعان رئيسيان من مرض السكري: النوع الأول (المعتمد على األنسولين) والنوع الثاني (غير المعتمد على األنسولين) نعم لا لا أعرف
الدواء أهم من الحمية والتمارين الرياضية للسيطرة على مرض السكري نعم لا لا أعرف
غالبا ما يسبب مرض السكري ضعف الدورة الدموية نعم لا لا أعرف
تلتئم الجروح بشكل أبطأ عند مرضى السكري نعم لا لا أعرف
يجب على مرضى السكر توخي الحذر عند قص أظافر االقدام نعم لا لا أعرف
يجب على المصاب بمرض السكر تطهير الجرح باليود والكحول نعم لا لا أعرف
ال تقل الطريقة التي أحضر بها طعامي أهمية عن األطعمة التي أتناولها نعم لا لا أعرف يمكن لمرض السكري أن يتلف كليتي نعم لا لا أعرف
يمكن لمرض السكري أن يضر عيني نعم لا لا أعرف
الرعشة والتعرق من عالمات ارتفاع السكر في الدم نعم لا لا أعرف
كثرة التبول والعطش من عالمات نقص السكر في الدم نعم لا لا أعرف
الجوارب المطاطية الضيقة جيدة لمرضى السكر نعم لا لا أعرف
يتكون النظام الغذائي لمرضى السكري في الغالب من أطعمة خاصة أمثلة األطعمة الخاصة: الخضروات والفواكه والحبوب ومنتجات األلبان الخالية من الدهون أو قليلة الدسم ومصادر البروتين ... من: األسماك أو الدجاج أو الديك الرومي بدون الجلد واللحوم الخالية من الدهون نعم لا لا أعرف
لرياضيون أقل عرضة لإلصابة بمرض السكري نعم لا لا أعرف
يشير معدل السكر من 100 إلى 125 مجم / ديسيلتر في الدم الصائم إلى أنك مصاب بمقدمات السكري نعم لا لا أعرف
يزيد سكري الحمل من مخاطر اإلصابة بالسكري من النوع 2 في المستقبل نعم لا لا أعرف
هل تعاني من داء السكري؟ نعم لا --> شكرا لمشاركتك. الذهاب الى section 13
ما هو نوع مرض السكري الذي تعاني منه؟ النوع الأول المعروف سابقا بمرض السكري الذي يصيب الاحداث أو الأطفال --> Skip to section 13 (شكرا لمشاركتك!) النوع الثاني المعروف سابقا بمرض السكري الذي يصيب البالغين او الكبار
معلومات عامة لمرضى السكري
منذ كم سنة وأنت مصاب بمرض بالسكري؟ يرجى ذكر عدد السنوات منذ تشخيص إصابتك بالمرض ، وليس تاريخ التشخيص. مثال اذا شخصت بالسكري من 8 سنوات اكتب فقط: 8
هل تتناول األنسولين لمرض السكري؟ نعم لا
هل تعرف ما هو مستوى مخزون السكر التراكمي لديك؟ المعروف بلقب HbA1C نعم لا
HBA1C مخزون السكر التراكمي
ما هو مستوى مخزون السكر التراكمي لديك؟ أقل من 5.7% بين 5.7% و 6.4% بين 6.4% و 7% بين 7% و 8% يساوي أكثر من 8%
السلوك
لا بأس في تناول الحلويات في بعض األحيان اعارض بشدة غير موافق محايد أوافق أوافق بشدة
لا بأس اذا نسيت تناول أدويتي في بعض الأيام اعارض بشدة غير موافق محايد أوافق أوافق بشدة
يجب أن أقوم بفحص دوري بناء على نصيحة الطبيب حتى لو كانت نسبة السكري لدّي تحت السيطرة اعارض بشدة غير موافق محايد أوافق أوافق بشدة لا بأس إذا لم أكن قادًرا على ممارسة الرياضة بقدر ما يطلب مني طبيبي ذلك ألنني أمارس تمريًنا كافًيا أثناء القيام بنشاطاتي اليومية اعارض بشدة غير موافق محايد أوافق أوافق بشدة
بشكل عام، أعتقد أن األشخاص الذين ال يحتاجون إلى تناول األنسولين لعالج مرض السكري لديهم مرض خفيف جًدا اعارض بشدة غير موافق محايد أوافق أوافق بشدة
بشكل عام، أعتقد أنه ال يوجد فائدة كبيرة في محاولة السيطرة الجيدة على نسبة السكر في الدم ألن مضاعفات مرض السكري ستحدث على أي حال اعارض بشدة غير موافق محايد أوافق أوافق بشدة
بشكل عام، أعتقد أن مرض السكري يؤثر تقريًبا على كل جزء من حياة الشخص المصاب بالسكري اعارض بشدة غير موافق محايد أوافق أوافق بشدة بشكل عام، أعتقد أن الحفاظ على نسبة السكر في الدم قريبة من المعدل الطبيعي يساعد في منع مضاعفات مرض السكري اعارض بشدة غير موافق محايد أوافق أوافق بشدة
بشكل عام، أعتقد أن ال داعي لألشخاص الذين يتم عالج مرض السكري لديهم من خالل نظام غذائي فقط للقلق بشأن التعرض للعديد من المضاعفات طويلة المدى اعارض بشدة غير موافق محايد أوافق أوافق بشدة
بشكل عام، أعتقد أن على كل شخص مصاب بالسكري تقريًبا أن يفعل كل ما يلزم للحفاظ على نسبة السكر في الدم قريبة من المعدل الطبيعي اعارض بشدة غير موافق محايد أوافق أوافق بشدة
بشكل عام، أعتقد أن التأثيرات العاطفية لمرض السكري ضئيلة جًدا اعارض بشدة غير موافق محايد أوافق أوافق بشدة بشكل عام ، أعتقد أن مرض السكري صعب ألن األشخاص المصابين به ال يتخلصون منه أبًدا اعارض بشدة غير موافق محايد أوافق أوافق بشدة
بشكل عام، أعتقد أن مرض السكري هو مرض خطير للغاية اعارض بشدة غير موافق محايد أوافق أوافق بشدة
بشكل عام، أعتقد أن اإلصابة بمرض السكري تغير نظرة الشخص إلى الحياة اعارض بشدة غير موافق محايد أوافق أوافق بشدة
بشكل عام، أعتقد أن األشخاص الذين يعانون من مرض السكري لن يستفيدوا كثيرًا من السيطرة الصارمة على نسبة السكر في الدم اعارض بشدة غير موافق محايد أوافق أوافق بشدة
بشكل عام، أعتقد أن على مرضى السكري أن يتعلموا الكثير عن المرض حتى يكونوا مسؤولين عن الاهتمام بأنفسهم اعارض بشدة غير موافق محايد أوافق أوافق بشدة
بشكل عام، أعتقد أنه من المحبط أن يعتني مرضى السكري بمرضهم اعارض بشدة غير موافق محايد أوافق أوافق بشدة
بشكل عام، أعتقد أن على األشخاص الذين يتناولون أدوية السكري عن طريق الفم أن يكونوا قلقين بشأن سكرالدم تماما مثل األشخاص الذين يتناولون أبر األنسولين اعارض بشدة غير موافق محايد أوافق أوافق بشدة
بشكل عام، أعتقد أن دعم األسرة واألصدقاء مهم في التعامل مع مرضى السكري اعارض بشدة غير موافق محايد أوافق أوافق بشدة
هل من المهم أن يراقب الشخص المصاب بالسكري ضغط دمه؟ اعارض بشدة غير موافق محايد أوافق أوافق بشدة
هل تعتقد أنه يجب عليك زيارة طبيبك بانتظام؟ اعارض بشدة غير موافق محايد أوافق أوافق بشدة
باإلمكان إيقاف تناول األدوية عند مراقبة مستوى السكري اعارض بشدة غير موافق محايد أوافق أوافق بشدة
يجب أن يخضع الشخص المصاب بالسكري لفحص شبكة العين بانتظام اعارض بشدة غير موافق محايد أوافق أوافق بشدة
يساعد خفض مستوى الدهون في التحكم بمستوى السكري من النوع الثاني اعارض بشدة غير موافق محايد أوافق أوافق بشدة
يشير اللجوء إلى األنسولين إلى مضاعفات في التحكم بمستوى السكري من النوع الثاني اعارض بشدة غير موافق محايد أوافق أوافق بشدة
يعد انخفاض السكر في الدم أخطر من ارتفاع السكر في الدم انخفاض السكر في الدم هو حالة يكون فيها مستوى السكر في الدم (الجلوكوز) أقل من الطبيعي، ارتفاع سكر الدم هو حالة .يكون فيها مستوى السكر في الدم )الجلوكوز( أعلى من المعدل الطبيعي اعارض بشدة غير موافق محايد أوافق أوافق بشدة
من الصعب السيطرة على نسبة السكر في الدم اعارض بشدة غير موافق محايد أوافق أوافق بشدة
تخاف من تناول الفاكهة والحلويات بسبب مخاوف من زيادة نسبة السكر في الدم اعارض بشدة غير موافق محايد أوافق أوافق بشدة
تخاف كثيرا من تناول وجبة (أو تقلل من الوجبات) بسبب مخاوف بشأن زيادة نسبة السكر في الدم بعد الاكل اعارض بشدة غير موافق محايد أوافق أوافق بشدة
الممارسة
هل تتناول أدوية لمرض السكر حسب إرشادات الطبيب؟ نعم لا
هل تمارس التمارين الرياضية بانتظام؟ نعم لا
هل تدخن (النرجيلة ، السيجارة اإللكترونية ..)؟ نعم لا
هل تتعرض للتدخين السلبي (هل تجلس بالقرب من اناس يدخنون السجائر أو النرجيلة)؟ نعم لا
هل مرضك (السكري) تحت السيطرة في الوقت الحاضر؟ تحت السيطرة: على سبيل المثال الحفاظ على مستويات السكر في الدم ضمن المستويات الصحية نعم لا لا أعلم
كم مرة تزور طبيبك؟ يوميا أسبوعيا شهريا كل شهرين الى 3 أشهر أكثر من 3 شهور
هل تتبع نظام غذائي منخفض السكر؟ نعم لا
هل تراقب وزنك؟ نعم لا
ل عانيت من انخفاض السكر في الدم بسبب اختيارات نمط الحياة غير المنتظمة؟-87 انخفاض السكر في الدم هو حالة يكون فيها مستوى السكر في الدم (الجلوكوز) أقل من الطبيعي نعم لا
هل عانيت من انخفاض السكر في الدم بين الوجبات، أو نقص السكر في الدم قبل النوم، أو نقص السكر في -88 الدم ليًلا؟ * انخفاض السكر في الدم هو حالة يكون فيها مستوى السكر في الدم (الجلوكوز) أقل من الطبيعي نعم لا
هل تقوم بفحص منتظم لمستوى الجلوكوز لديك أثناء النهار؟ نعم لا
هل تراقب ضغط الدم بانتظام؟ نعم لا كم مرة قمت بزيارة طبيب عيون خالل الخمس سنوات الماضية؟ 0 1 2 3 4 5 أكثر من 5
هل تتوخى الحذر أثناء تقليم الأظافر؟ نعم لا
هل تعرف كيف تتصرف في حالة نسيت تناول أدوية السكري؟ نعم لا
هل تسأل طبيبك أو الصيدلي عن توصيات أو نصائح بخصوص مرض السكر لديك؟ نعم لا
هل تزور طبيبك بانتظام؟ نعم لا
لماذا ال تزور طبيبك بانتظام؟ لا تستطيع تحمل الكلفة غياب دعم الآسرة لا تعتقد أنه مهم لا تجد الوقت يكفيك فحص مستويات السكر باستخدام جهاز قياس السكر في المنزل لم تكن تعلم أن المتابعة المنتظمة ضرورية أسباب اخرى
هل دخلت المستشفى في الثالثين يوًما الماضية بسبب مضاعفات مرض السكري؟ نعم لا
